# A New Method of Laryngoscopy—Animal Grafts

**Published:** 1869-02-15

**Authors:** 


### FOREIGN ITEMS.

A New Method of Laryngoscopy.—Dr. Voltilini, in an article just published in the Berlin *Medicat Weekly*, affirms that the epiglottis and the interior of the thyroid cartilage can be seen without the aid of the laryngoscopic mirror. It is not necessary to press upon the tongue with the tongue-depressor, which obstructs the view, but to extend the tongue firmly outside of the mouth; at the same time it is necessary to press externally upon the thyroid cartilage with the fingers of the hand which holds the tongue, and to push the epiglottis forward by means of a little instrument designed for the purpose. The author maintains that he has been able in this manner, to see the thyroid cartilage, and that it is possible thus to explore the interior of the thyroid body, in an appropriate subject.—*Gazette Médicale.*

Animal Grafts—Dr. Montegazza refers to some interesting experiments, which he had made, upon animal grafting, and the artificial production of cells, and which he had recorded in a volume published in Milan, in 1865. In this work he investigated the changes which supervene in a tissue when it is transplanted, without having lost its vital properties, into the substance of an organism still possessing life. He applies the organs removed to an animal living in conditions most favorable to the preservation of their physiological properties; he interrupts only the course of the nerves and the direct communication of the vessels, he affects for them a reciprocal combat by means of the plasma, and he thus preserves the natural temperature, humidity, etc. By pursuing this method he has been enabled to observe in some tissues a very rapid fatty degeneration; in others he has established the preservation of physiological nutrition, whilst others still have continued to grow even beyond their normal limits; finally, in extremely rare cases, he has been able to demonstrate that not only the nutritive faculty but the function, even, of the organ was preserved. The stomach has furnished him with the most striking example of the fact. He had observed that this viscus, isolated from all nervous and vascular communication, could preserve, during almost a month, the faculty of secreting mucus and of digesting; all the stomachs transplanted were distended by mucus, and in some it seemed that this accumulation would burst the walls. He effected artificial digestion as well in stomachs transplanted upon the same animal, as in stomachs transferred from one animal to another; but in the first, the digestive force was more energetic, and, what was very remarkable, it was even much more energetic than in two stomachs which had just been removed from living and well nourished frogs.

Desiring to renew these experiments, but prevented by other occupations, M. Marchioli, his assistant, assumed the charge of conducting them. Subjoined is an analysis of three of these experiments. In the first the stomach was removed from a living frog, on the 8th of January, and after having received a double ligature, at the cardiac and and pyloric orifices, it was placed in the abdominal cavity of another, very robust frog. Cicatrization occurred promptly: the frog continued in good health up to the 22d of January, when it died. Upon opening the abdominal cavity the engrafted stomach was found adhering tenaciously to the peritoneum, near the greater curvature of the stomach proper of the frog. Both were empty and contained but little mucus. The mucus of the two stomachs was scraped, and the pulp which resulted therefrom was placed in two little glasses, into which was poured five cubic centimetres of distilled water and a drop of hydrochloric acid.

Next the digestive power of the two stomachs was tested by placing them in contact with cubes of boiled white of egg, and a difference of forty-eight hours in favor of the grafted stomach was determined. The second and the third experiments were but repetitions of the first, and were followed by analogous results.

